# Differential microRNA Expression in Porcine Endometrium Involved in Remodeling and Angiogenesis That Contributes to Embryonic Implantation

**DOI:** 10.3389/fgene.2019.00661

**Published:** 2019-07-26

**Authors:** Linjun Hong, Ruize Liu, Xiwu Qiao, Xingwang Wang, Shouqi Wang, Jiaqi Li, Zhenfang Wu, Hao Zhang

**Affiliations:** ^1^National Engineering Research Center for Breeding Swine Industry, College of Animal Science, South China Agricultural University, Guangzhou, China; ^2^Guangdong Provincial Key Laboratory of Agro-animal Genomics and Molecular Breeding, College of Animal Science, South China Agricultural University, Guangzhou, China; ^3^Stanley Center for Psychiatric Research, Broad Institute of MIT and Harvard, Cambridge, MA, United States

**Keywords:** porcine, endometrium, microRNAs, differential expression, implantation

## Abstract

**Background:** In western swine breeds, up to 30% of embryonic losses occur during early pregnancy, and the majority of embryonic losses happens during implantation. In this period, maternal recognition of pregnancy begins to occur and blastocysts undergo dramatic morphologic changes. As with other species, changes in the uterine environment plays an important role in the process of embryo implantation in pigs. Erhualian (ER) pigs, one of the Chinese Taihu swine breeds, are known to have the highest litter size in the world. Experiments demonstrated that the greater embryonic survival on gestation day (GD) 12 in Chinese Taihu pigs is one important factor that contributes to enhanced litter size. This is largely controlled by maternal genes. In this study, endometrial samples were collected from pregnant Landrace×Large Yorkshire (LL) sows (parity 3) and ER sows (parity 3) on GD12 and the expression profiles of microRNAs (miRNAs) in the endometrium were compared between ER and LL using miRNA-seq technology.

**Results:** A total of 288 miRNAs were identified in the pig endometrium, including 202 previously known and 86 novel miRNAs. The Kyoto Encyclopedia of Genes and Genomes pathway analysis revealed that highly abundant miRNAs might affect endometrial remodeling. Comparison between LL and ER sows revealed that 96 known miRNAs were differentially expressed between the two groups (including 78 up-regulated and 18 down-regulated miRNAs in ER compared to LL). Bioinformatics analysis showed that the target genes of some differentially expressed miRNAs were involved in pathways related to angiogenesis, proliferation, apoptosis, and tissue remodeling, which play critical roles in implantation by regulating endometrial structural changes and secretions of hormones, growth factors, and nutrients. Furthermore, the results demonstrated that insulin-like growth factor-1 protein expression was directly inhibited by miR-206. The lower expression of miR-206 in ER compared to LL might facilitate the angiogenesis of the endometrium during embryo implantation.

**Conclusions:** The identified miRNAs that are differentially expressed in the endometrium of ER and LL pigs will contribute to the understanding of the role of miRNAs in embryonic implantation and the molecular mechanisms of the highest embryonic survival in Chinese ER pigs.

## Introduction

Litter size has a great impact on the profitability of swine production. Prenatal mortality is the major limitation for increasing the litter size in pigs. Up to 30% of conceptuses are spontaneously lost during early pregnancy, especially on gestation days (GD) 11 to 13 (Scofield et al., 1974; Pope and First, 1985; Zavy and Geisert, 1994; Wilson et al., 1999). In contrast to rodents and primates, pigs have an extended period of preimplantation. From GD4 to GD12, the developing conceptuses undergo speedy morphologic changes (from spherical to tubular to filamentous forms) and migrate freely in the uterine cavity. On GD15, filamentous conceptuses grow to 800 to 1,000 mm in length and begin to attach to luminal uterine epithelium (LE) (Geisert et al., 1982; Bazer and Johnson, 2014). Thus, during protracted preimplantation, the requirement for nutrients of conceptuses is mainly dependent on uterine secretions, including glucose, amino acids, ions, enzymes, growth factors, hormones, growth factors, and other substances termed as histotroph (Spencer et al., 2006).

Chinese Taihu pigs, including Erhualian (ER), Meishan, and Fengjing breeds, are highly prolific. ER pigs are known to have the biggest litter size record in the world (Zhang, 1986). Meishan pigs were exported to western countries in the early 1980s and have been studied for more than 30 years to explore the mechanism of prolificacy. Studies found that the greater embryonic survival on GD11 to GD12 in Chinese Taihu pig is the most important factor contributing to enhanced litter size, and this is controlled primarily by maternal genes (Haley et al., 1995). At this stage, substantial changes occur in the conceptus-uterine interface, including morphologic changes in the conceptus, the onset of synthesis of estradiol by the conceptus, and the appropriate physiologic adjustments of uterus (Bazer and Johnson, 2014). Examination of individual embryos in Meishan pigs found that embryo survival was 108.1% on GD11 and 93.3% on GD12; however, in Landrace×Large Yorkshire (LL) pigs, embryo survival was 89.1% on GD11 and 49.9% on GD12 (Ashworth et al., 1997). Other studies also demonstrated that, from GD11 to GD12, the embryonic survival rate in Chinese Taihu pigs was significantly higher than in western pigs (Bazer et al., 1988; Christenson et al., 1993). Thus, it is worth further studying the molecular mechanisms underlying the differences in uterine environment changes between Chinese Taihu and western pigs.

microRNAs (miRNAs) are short (20–25 nt), endogenous, conserved, non-coding RNA molecules that play wide biological roles in transcription and translation (Carrington and Ambros, 2003; Bartel, 2004). miRNAs typically interact with target mRNAs by base pairing and destabilize or degrade their complementary mRNA (Wu et al., 2006). They have been shown to participate in the regulation of various physiologic processes, including cellular proliferation, differentiation, apoptosis, angiogenesis, embryonic development, and reproduction control (Laurent, 2010; Nicoli et al., 2012; Rosenbluth et al., 2013). A large number of miRNAs have been shown to be associated with embryo implantation in humans and mice, such as the regulation of endometrial receptivity (Altmäe et al., 2013) and endometrial stromal cell differentiation (Qian et al., 2009), participating in human pregnancy and parturition (Montenegro et al., 2009). In pigs, Su et al. and Liu et al. reported that miRNAs play roles in porcine placental growth and functions (Su et al., 2010; Liu et al., 2015). In addition, Wessels et al. investigated the expression of miRNAs on both sides of the maternal-fetal interface in the model of implantation failure and spontaneous fetal loss in pigs and identified miRNAs that might contribute to fetal loss (Wessels et al., 2013). Thus, taken together, these results indicate the importance of miRNAs in pig reproduction. In this study, the expression profiles of miRNAs in the sow endometrium on GD12 were compared between ER and LL pigs using sequencing technology. Differentially expressed miRNAs (DEMs) were identified and miRNAs involved in reproduction were analyzed by bioinformatic analysis and experiments. Collectively, these results will help better understand the role of miRNAs in embryonic survival during implantation.

## Materials and Methods

### Tissue Collection

All of the experiments involving animals were conducted according to animal ethics guidelines and approved by the Animal Care and Use Committee of South China Agricultural University (Guangzhou, China). LL and ER sows were obtained from the breeding pig farm of Guangdong Wen’s Foodstuffs Group Co., Ltd. (Yunfu, China). Three LL sows (parity 3) and three ER sows (parity 3) were checked for estrus twice daily and artificially inseminated at the onset of estrus (day 0) and again 12 h later. After the sows were slaughtered at a local slaughterhouse on GD12, the uteri were removed rapidly and transported in an icebox to the laboratory. Pregnancy was confirmed by the presence of apparently normal filamentous conceptuses in uterine ﬂushings. Endometrial samples were collected and stored at −80°C for RNA extraction.

### RNA Extraction and Small RNA (sRNA) Sequencing

Total RNA was extracted from six endometrial samples using TRIzol (Invitrogen, Carlsbad, CA, USA) according to the manufacturer’s instruction. RNA purity was quantified using NanoDrop ND2000 spectrophotometer at 260 and 280 nm (Thermo Fisher Scientific, Wilmington, MA, USA), and RNA integrity was verified using an Agilent 2100 Bioanalyzer (Agilent Technologies, Palo Alto, CA, USA). The OD260/OD280 ratios of all the samples were greater than 1.8, and the RIN values were greater than 8. Equal RNA quantities from the endometria of three pigs from the LL and ER groups were pooled. sRNA Illumina sequencing was conducted as follows: ∼10 μg total RNA was size fractionated by Novex 15% TBE-Urea gel and RNA fragments between 18 and 30 bases in length were isolated. The purified sRNAs were then ligated with the 5′-adapter. To remove unligated adapters, the ligation products (36–50 bases in length) were gel purified on Novex 15% TBE-Urea gel. Subsequently, the RNA fragments with the adapter at the 5′-end were ligated with 3′-adapters. After gel purification on Novex 10% TBE-Urea gel, RNA fragments with adapters at both ends (62–75 bases long) were reverse transcribed. Reverse transcription-polymerase chain reaction (RT-PCR) was used to create cDNA constructs based on the sRNA ligated with the 5′- and 3′-adapters. This protocol gel purifies the amplified cDNA construct in preparation for loading on the Illumina Cluster Station. The cDNAs were amplified using the appropriate PCR cycles to produce sequencing libraries. Sequencing was carried out at BGI-Shenzhen, China.

### Sequence Analysis

First, raw data (raw reads) were processed by custom Perl and Python scripts, raw reads contain poly-A/T/G/C, poly-N, with 5′-adapter contaminants, without 3′-adapter, or the insert tag, and low-quality reads were filtered to get clean data. Subsequently, clean reads ≥18 nt were chosen as sRNA tags and mapped to reference sequence by Bowtie (Langmead et al., 2009) without mismatch to analyze their expression and distribution on the reference. Third, miRBase (release 20.0) was used as reference, and srna-tools-cli and modified software mirdeep2 (Friedländer et al., 2011) were used to obtain the potential miRNA and draw the secondary structures. The available software mirdeep2 (Friedländer et al., 2011) and miREvo (Wen et al., 2012) were integrated to predict novel miRNAs by exploring the secondary structure. At the same time, custom scripts were used to obtain the identified miRNA counts as well as base bias on the first position with certain length and on each position of all identified miRNAs, respectively.

### Identification of DEMs

The procedures that determine the DEMs between LL and ER groups are shown below:

miRNA expression levels were estimated by transcript per million (TPM) with the following criteria (Wagner et al., 2012): Normalization formula: Normalized expression = Actual miRNA count/Total count of clean reads*1,000,000. miRNAs with a normalized expression level of less than 1 in each of the two libraries and miRNAs with an estimated probability value of less than 0.95 were removed. The fold change in the expression level and the *P* value between two libraries were calculated from the normalized expression using the following formulas, respectively:

Fold change formula: Fold change = log_2_(ER/LL)

*P*-value formula:

$$p(x|y)={\left(\frac{N_{2}}{N_{1}}\right)}^{y}\frac{(x+y)!}{x!y!{\left(1+\frac{N_{2}}{N_{1}}\right)}^{\left(x+y+1\right)}}$$

*N*_1_ and *x* represent the total count of clean reads and the normalized expression level of a given miRNA in an sRNA library of LL endometrial tissue sample, respectively. *N*_2_ and *y* represent the total count of clean reads and the normalized expression level of a given miRNA in an sRNA library of ER endometrial tissue sample, respectively (Audic and Claverie, 1997). Raw *P* values were converted to adjusted *P* values using the Benjamini–Hochberg false discovery rate (Benjamini and Hochberg, 1995). The adjusted *P* < 0.05 and |log_2_(fold change)| > 1 were set as thresholds for significantly differential expression by default.

### Target Gene Prediction and Functional Analysis of DEMs

The prediction of the target genes of miRNAs was performed by RNAhybrid and TargetScan. Overlapping target genes were selected for further analysis. To reveal the target genes’ potential biological functions and identify the main pathways targeted by the gene candidates, Gene Ontology (GO) and Kyoto Encyclopedia of Genes and Genomes (KEGG) pathway analyses were performed as described previously (Zhang et al., 2013). Based on the GO and KEGG database, the hypergeometric test was preformed to identify significantly enriched GO terms (*Q* < 0.05) and classify the pathway category (Boyle et al., 2004). The network of pathways based on the GO and KEGG database was constituted by ClueGO, which is a plugin in Cytoscape (http://www.cytoscape.org/).

### Validation of miRNA Expression via Stem-Loop Quantitative RT-PCR (qRT-PCR)

The sRNA-seq results were validated using RNA samples from the LL (*n* = 3) and ER (*n* = 3) groups by the stem-loop qRT-PCR method. A total of 16 miRNAs were selected for qRT-PCR validation. The mature miRNA and primer sequences are available in **Supplementary Table S1**. Briefly, for RT-PCR, the Revert Aid™ First Strand cDNA Synthesis Kit (Promega, Fitchburg, WI, USA) was adopted according to the manufacturer’s instructions. Then, RT-PCR was performed with SYBR^®^ Premix Ex Taq™ (Toyobo) on ABI PRISM^®^ 7500 Sequence Detection System. Porcine U6 snRNA was used as an internal control and all reactions were run in triplicate. PCR profiles were one cycle at 95°C for 5 min followed by 40× (95°C for 15 s, 65°C for 15 s, and 72°C for 32 s). The relative expression levels were calculated using the 2^−ΔΔCt^ method. Fold change (log_2_ ratio) was used to show the differential expression of miRNA in LL and ER.

### Dual-Luciferase Reporter Assays

For luciferase reporter experiments, the pmirGLO dual-luciferase reporter vector (Promega) housing the 3′-untranslated region (UTR) of insulin-like growth factor-1 (IGF-1), which was *Xho*I and *Xba*I cloned to the 3′-end of the *Renilla* gene, was used to examine the effect of miR-206 on *Renilla* production. IGF-1 3′-UTR vector (pmirGLO-IGF1) containing the miR-206 binding site (CATTCC) was constructed by RT-PCR using specific primers (forward primer 5′-CCGCTCGAGCAGGAAACAAGAACTACAG-3′ and reverse primer 5′-GCTCTAGACAACAGCAATCTACCAACT-3′). Meanwhile, IGF-1 3′-UTR-Mutant vector (pmirGLO-IGF1-Mutant) with a mutated miR-206 binding site (GTAAGG) was also constructed. The miR-206 mimics and its mutant mimics (miR-206_mut) were designed and synthesized by GenePharma Biotech Co. (Shanghai, China). In the dual-luciferase assays, PK15 cells were cultured in DMEM complete medium (Hyclone, Logan, UT, USA) and then plated onto a 96-well plate. The miR-206 mimics, mutant miR-206mimics, or negative control (NC) were co-transfected into cells with 3′-UTR dual-luciferase vector using Lipofectamine 2000 (Invitrogen, Shanghai, China). Cells were collected 24 h after transfection, and assayed with the Dual-Luciferase Reporter Assay System (Promega). Three replicates were performed for each transfection.

### Lentivirus Preparation and Administration

The pri-miR-206 expression lentivirus vector (H1-MCS-CMV-EGFP) and the NC lentivirus vector were purchased from GenePharma Biotech. Virus titration and infection efficiency were measured by the fluorescence method as lentiviral vectors expressed enhanced green fluorescent protein in infected cells. According to the results of a preliminary experiment, the titer of lenti-pri-miR-206 used for experiments was 1 × 10^7^ TU/ml. The lentivirus vectors were transfected into porcine skeletal muscle satellite cells (SCs) with a titer of 1 × 10^7^ TU/ml in the presence of polybrene (5 µg/ml). Cells were collected 72 h after transfection, and total RNA and protein were extracted for further experiments.

### Western Blot Analysis

Protein lysates were generated using the mammalian protein extraction reagent RIPA (Beyotime, Shanghai, China). The concentration of extracted total protein from each sample was calculated using the BCA Protein Assay Kit (Thermo Pierce, Rockford, IL, USA). The equivalent protein for each sample was loaded into a 10% sodium docedyl sulfate-polyacrylamide gel electrophoresis and fractionated, and the denatured proteins were subsequently transferred from gel to a polyvinylidene fluoride membrane (Millipore, Billerica, MA, USA) by a Mini-PROTEAN Tube Cell instrument (Bio-Rad, Hercules, CA, USA). The membranes were incubated with antibodies (IGF-1, ab9572, Abcam; glyceraldehyde 3-phosphate dehydrogenase, ab8245, Abcam) overnight at 4°C and then with horseradish peroxidase-conjugated goat anti-rabbit secondary antibody for 1 h at room temperature. The enhanced chemiluminescence substrate (Beyotime) was used to visualize the band, and a picture was captured by an imaging system (UVP, Upland, CA, USA). Finally, the quantification analysis was performed by ImageJ 1.45 software (NIH Image).

### Statistical Analysis

Data from the results of qRT-PCR, dual-luciferase reporter assays, and Western blot analysis were analyzed using SPSS version 18.0 (SPSS, Inc., Chicago, IL, USA). Paired *t* tests and two-way analyses of variance were performed to analyze the relative expression of miRNAs, the luciferase activity, and the intensity of the protein band in Western blot analysis. *P* < 0.05 was considered statistically significant.

## Results

### Overview of the Squences Generated by Illumina Sequencing

**Figure 1:**
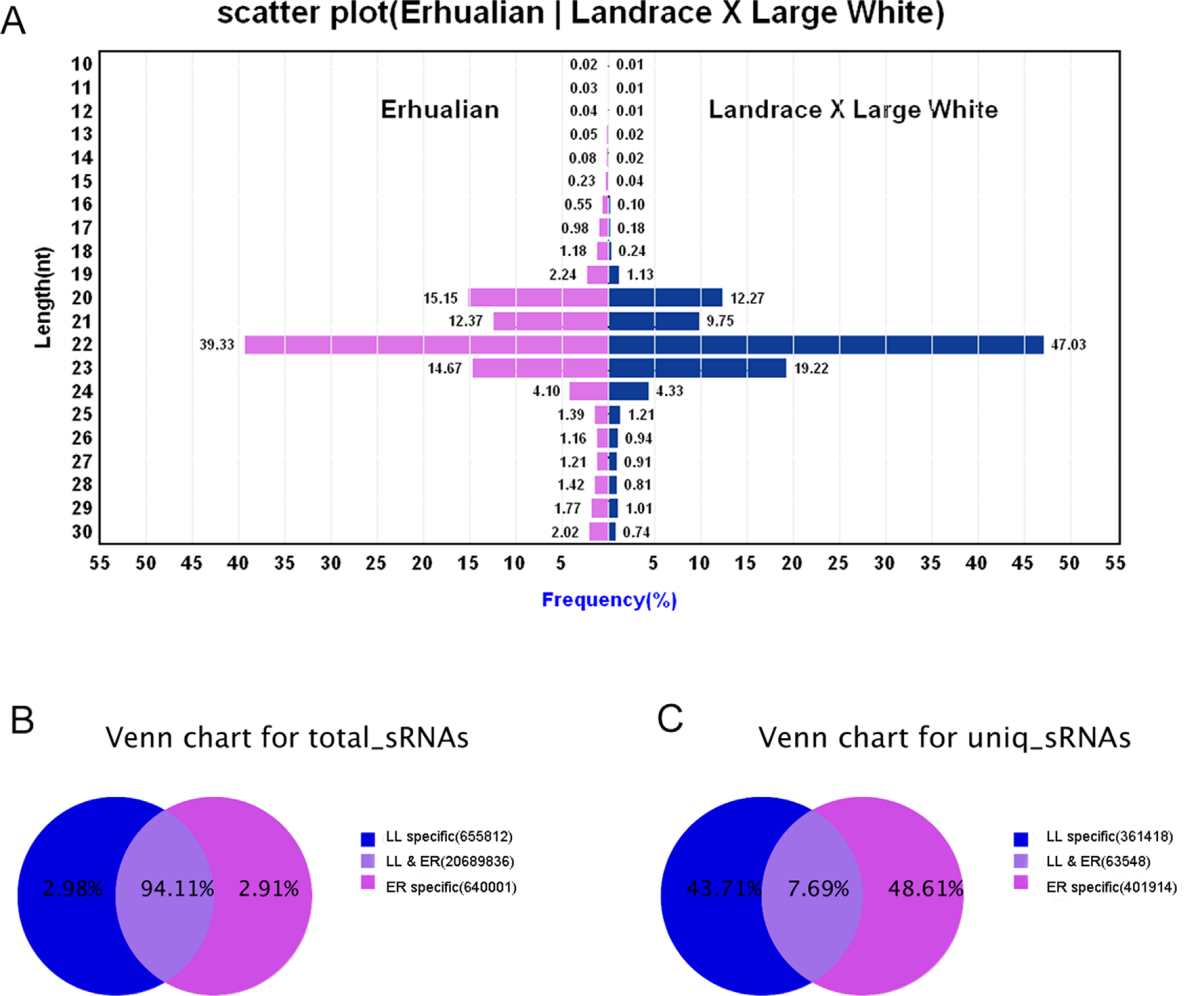
Overview of the sequences generated by Illumina sequencing. **(A)** Sequence length distributions of the two libraries. The length distributions peaked at 22 nt, which is expected for miRNAs’ length. **(B)** Number of total sRNA tags between the two libraries. **(C)** Number of unique sRNA tags between the two libraries.

sRNA libraries were generated from a total of six samples from ER and LL sows on GD12. After removing low-quality reads and adaptor sequences, a total of 9,104,438 and 12,881,211 clean reads were obtained from ER and LL samples, respectively. The sRNA annotation is presented in **Supplementary Table S2**. The results of sRNA annotation showed that known miRNAs accounted for 48.64% and 56.96% of the total clean reads in ER and LL, respectively (**Supplementary Table S2**). The distribution of sequence lengths was similar between ER and LL libraries (**Figure 1A**). The number of 20 to 23 nt sequences was significantly greater than that of shorter or longer sequences, and almost half of the sequences in LL (47.03%) and 39.33% sequences in ER are 22 nt.

In total sRNA reads, 20,689,836 common sequences were obtained in LL and ER, accounting for 94.11% of the total sequence reads in the two libraries (**Figure 1B**). In unique sRNA reads, 63,548 common sequences were obtained in LL and ER, accounting for 7.69% of the total reads in the two libraries (**Figure 1C**), and 361,418 (43.71%) and 401,914 (48.61%) specific sequences were obtained from LL and ER, respectively (**Figure 1C**).

#### Sequence Variants and Editing of Bases in the Seed Region of miRNAs

**Figure 2:**
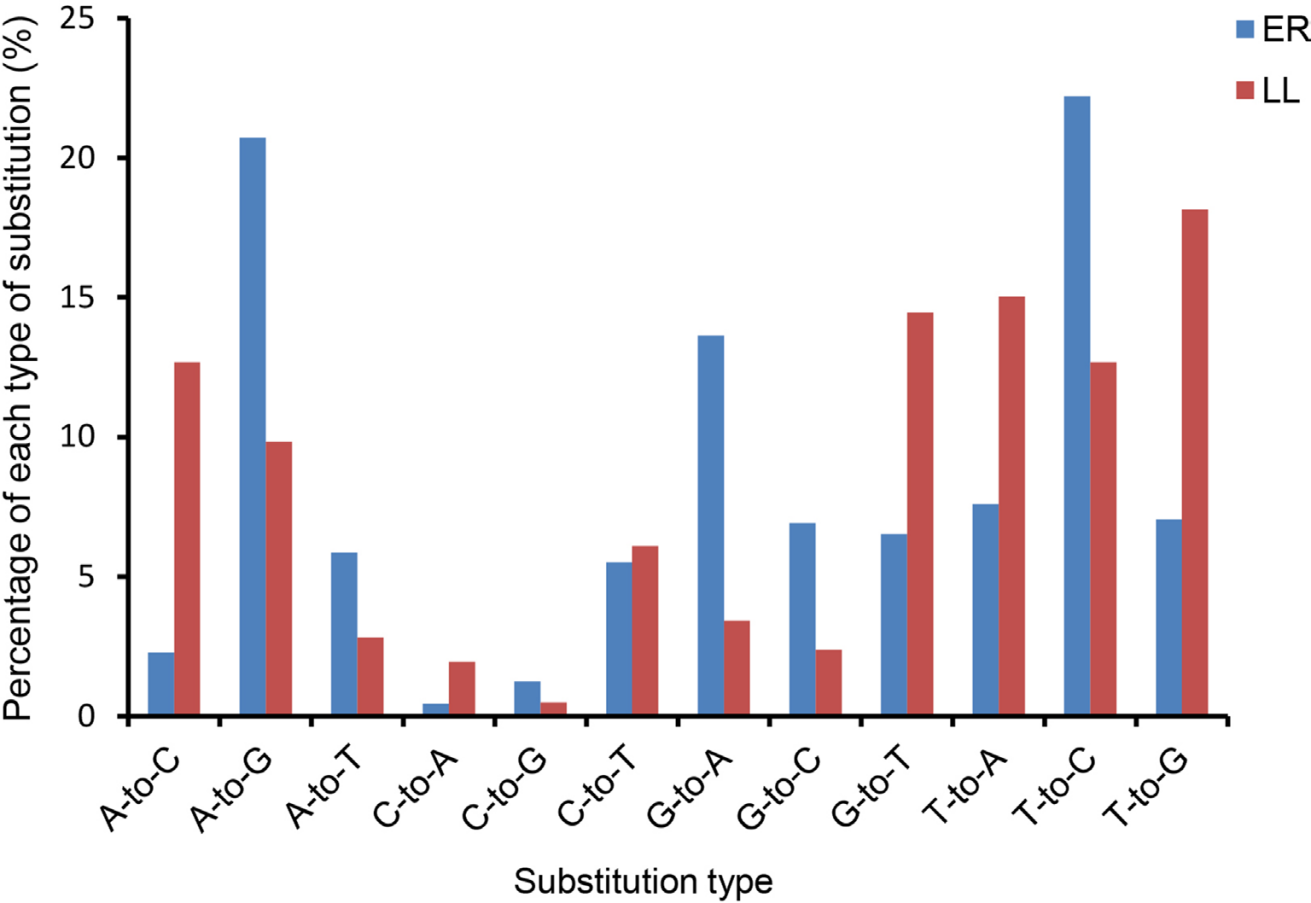
Histogram displaying the single nucleotide substitution in the miRNA seed region sequence when aligning un-annotated sRNA tags with porcine mature miRNAs from miRBase 20.0.

Sequencing data analysis revealed that the majority of identified miRNAs showed length and sequence heterogeneity in the porcine endometrial tissue. The length variations occurred largely in the 3′-end of miRNAs, mainly in the form of terminal reductions or additions of nucleotides. In ER, miR-128, miR-187, miR-18b, miR-190, miR-196a, miR-206, miR-215, miR-2476, miR-326, miR-338, miR-676, and miR-758 had variants only at the 3′-end, whereas, in LL, miR-105, miR-129b, miR-149, miR-153, miR-190, miR-208b, miR-216, miR-450a, miR-450c-5p, miR-499-5p, miR-503, and miR-95 had variants only at the 3′-end. In addition, 11 and 10 miRNAs in the ER and LL libraries, respectively, were mutated by only one nucleotide in the 5′-end, but they had several 3′-end variants (**Supplementary Tables S3** and **S4**). Similarly, previous studies also revealed the length variations of miRNAs in other porcine tissues (Li et al., 2010; Nielsen et al., 2010; Li et al., 2011). Such variants might be from altered miRNA processing, prioritized degradation at miRNA ends, or post-transcriptional modifications, including RNA editing (Aravin and Tuschl, 2005). These end-sequence variations are interesting as they may allow miRNA variants to play different roles by influencing the miRNA-target mRNA hybrid duplex structure (Jazdzewski et al., 2009).The nucleotides at positions 2 to 8 of a mature miRNA are known as the seed region. The seed region binds to a target site in the 3′-UTR of the target mRNA by complementarities and is highly conserved. The target of an miRNA may alter due to change in the nucleotides in the seed region. Editing of bases in the seed region of miRNAs has been reported to occur frequently (Kawahara et al., 2007; Liu et al., 2008). In the present analysis, miRNAs that might have seed editing can be distinguished by matching unannotated sRNA with porcine mature miRNAs from miRBase 20.0. Forty-nine and 62 mature miRNAs in ER and LL had a single nucleotide substitution in the seed region, respectively (**Supplementary Table S5**). The observed occurrence for each possible substitution is summarized in **Supplementary Table S5** for ER and LL samples, respectively. In ER, the most frequent substitutions were T-to-C (22.2%), A-to-G (20.7%), and G-to-A (13.6%), whereas, in LL, the most frequent substitutions were T-to-G (18.1%), T-to-A (15.0%), and G-to-T (14.5%; **Figure 2**). Although the most frequent substitutions were different between ER and LL libraries, C-to-A (0.4% in ER; 2.%) and C-to-G (0.4% in ER; 2.%) were the substitutions with the lowest frequency in both ER and LL libraries. In porcine adipose tissue samples, similar results were also reported (Li et al., 2011). Interestingly, abundant miRNAs (ssc-let-7a, ssc-mir-143, ssc-let-7f, ssc-mir-21, and ssc-mir-378) also had higher editing probability (**Supplementary Table S5**). This indicates that highly expressed miRNAs targeted more genes.

### Expression Profiling of miRNAs

In the second part of the present analysis, the global expression profile of endometrial miRNAs on GD12 in LL and ER pigs was determined. A total of 288 miRNAs were identified in the pig endometrium, including 202 known miRNAs and 86 novel miRNAs (**Supplementary Table S6**). Among the known miRNAs, 200 miRNAs were co-expressed in LL and ER and 2 miRNAs (ssc-miR-124a and ssc-miR-450c-3p) were specifically expressed in ER. Among the novel miRNAs, 38 miRNAs were co-expressed in LL and ER and 19 and 28 miRNAs were specifically expressed in LL and ER, respectively.

**Figure 3:**
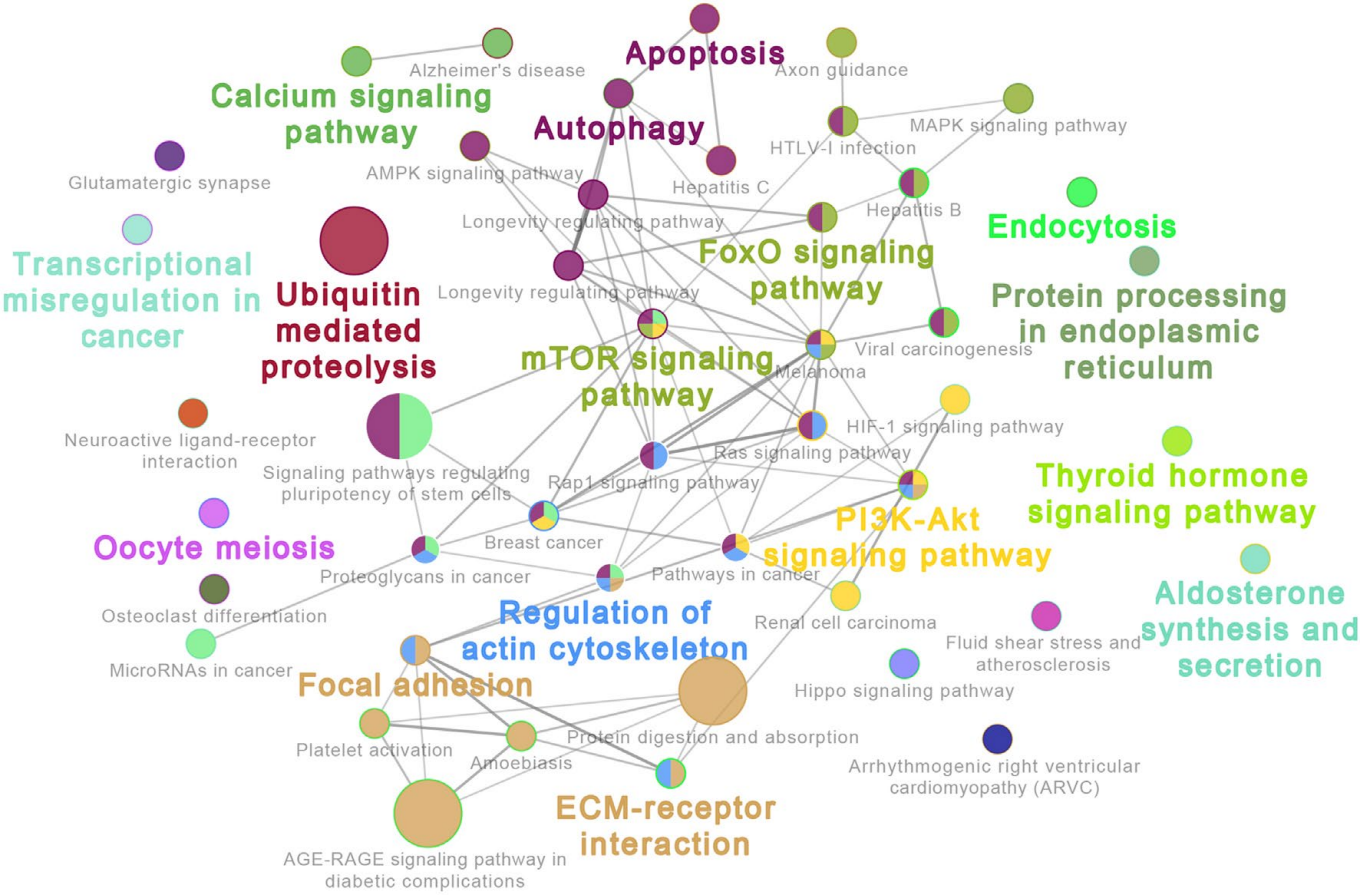
ClueGo network of pathways. Each node represents a pathway. The enrichment significance of pathway is reflected by the size of the nodes. Node color represents the class that they belong to. Mixed coloring means that the specific node belongs to multiple classes.

The 20 most highly expressed miRNAs in LL and ER libraries are listed in **Table 1**. Among them, 14 highly expressed miRNAs were the same in LL and ER. Thus, the predicted target genes of 14 common miRNAs were chosen for functional analysis. GO analysis (ClueGo network of GO terms) showed that they were mainly involved in the “cellular protein metabolic process,” “regulation of macromolecule biosynthetic process,” and “anatomical structure morphogenesis” (**Supplementary Figure S1**). The KEGG pathway analysis (ClueGo network of pathways) indicated that the predicted target genes were mainly enriched in “Apoptosis,” “Autophagy,” “Ubiquitin-mediated proteolysis,” “Longevity-regulating pathway,” “AMPK signaling pathway,” “Regulation of actin cytoskeleton,” “Focal adhesion,” “ECM-receptor interaction,” “Rap1 signaling pathway,” “FoxO signaling pathway,” “mTOR signaling pathway,” and “MAPK signaling pathway” (**Figure 3**).

**Table 1:** Top 20 miRNAs in LL and ER.

miRNA-name	Average TPM in LL	Rank in LL	miRNA-name	Average TPM in ER	Rank in ER
ssc-miR-143-3p	2208321	1	ssc-miR-143-3p	1266790	1
ssc-let-7a	1289290	2	ssc-let-7a	836090	2
ssc-miR-21	519081	3	ssc-miR-21	509408	3
ssc-let-7f	467190	4	ssc-let-7f	317357	4
ssc-miR-30a-5p	313695	5	ssc-miR-30a-5p	92442	9
ssc-miR-148a	290344	6	ssc-miR-148a	31042	18
ssc-miR-10a	242124	7	ssc-miR-10a	112910	8
ssc-miR-10b	240246	8	ssc-miR-10b	133656	6
ssc-let-7c	224940	9	ssc-let-7c	129079	7
ssc-miR-378	184397	10	ssc-miR-378	213166	5
ssc-miR-30d	138588	11	ssc-miR-30d	63006	10
ssc-miR-34c	102496	12	ssc-miR-34c	13563	31
ssc-miR-140*	88368	13	ssc-miR-140*	21112	22
ssc-miR-103	79372	14	ssc-miR-103	32711	17
ssc-let-7g	78468	15	ssc-let-7g	38160	16
ssc-miR-126	45626	16	ssc-miR-126	23587	21
ssc-miR-1	44500	17	ssc-miR-1	4031	57
ssc-miR-191	44154	18	ssc-miR-191	9654	36
ssc-miR-30e-5p	43404	19	ssc-miR-30e-5p	27545	20
ssc-miR-206	41179	20	ssc-miR-206	233	128
ssc-miR-26a	40215	22	ssc-miR-26a	47934	11
ssc-miR-101	34754	23	ssc-miR-101	41815	12
ssc-miR-196b-5p	30481	27	ssc-miR-196b-5p	27714	19
ssc-miR-199a*	21283	32	ssc-miR-199a*	39465	13
ssc-miR-199a-3p	21236	33	ssc-miR-199a-3p	39427	14
ssc-miR-199b*	21236	34	ssc-miR-199b*	39426	15

The list shows the top 20 abundant miRNAs in LL and ER, respectively.

The star is part of the miRNA name.

### Comparative Analysis of DEMs Between ER and LL

**Figure 4:**
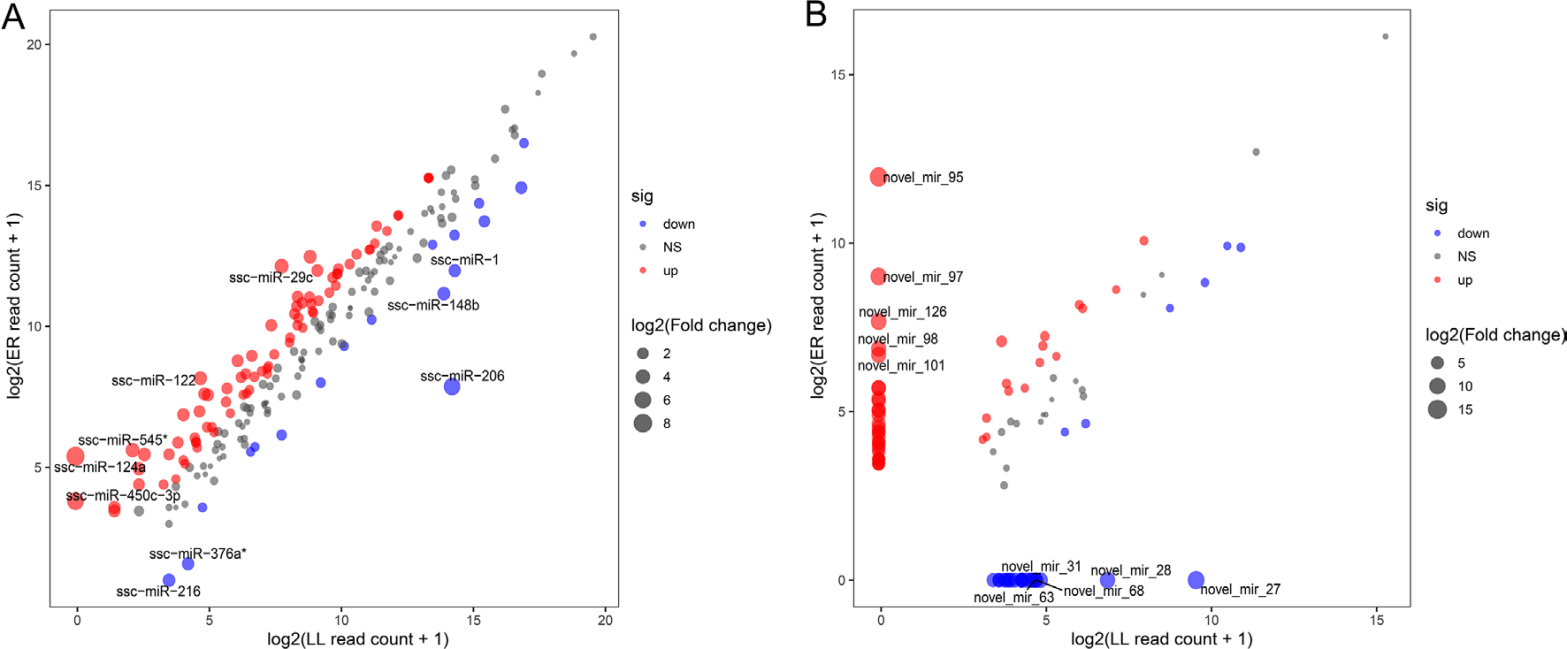
Differential expression of porcine known **(A)** and novel **(B)** miRNAs between ER and LL. Each point in the figure represents the log_2_(ER/LL read count + 1) of an miRNA. Red points represent miRNAs with log_2_(ER/LL) > 1 and adjusted *P* < 0.05, blue points represent miRNAs with log_2_(ER/LL) < −1 and adjusted *P* < 0.05, and green points represent miRNAs with 1 > log_2_(ER/LL) > −1. The size of points shows the value of log_2_(ER/LL).

These DEMs between LL and ER libraries are listed in **Supplementary Table S7**, and in total, 96 known and 68 novel significantly DEMs were identified between LL and ER groups. Of the 96 differentially expressed known miRNAs, 78 were up-regulated and 18 were down-regulated in ER compared to LL (**Figure 4A**). miR-206 ranked the top [fold change log_2_(ER/LL) = −6.96] among DEMs that were expressed in both LL and ER. Of the differentially expressed novel miRNAs, 43 were up-regulated and 25 were down-regulated in ER than in LL (**Figure 4B**).

### Validation of Sequencing Results by qRT-PCR

**Figure 5:**
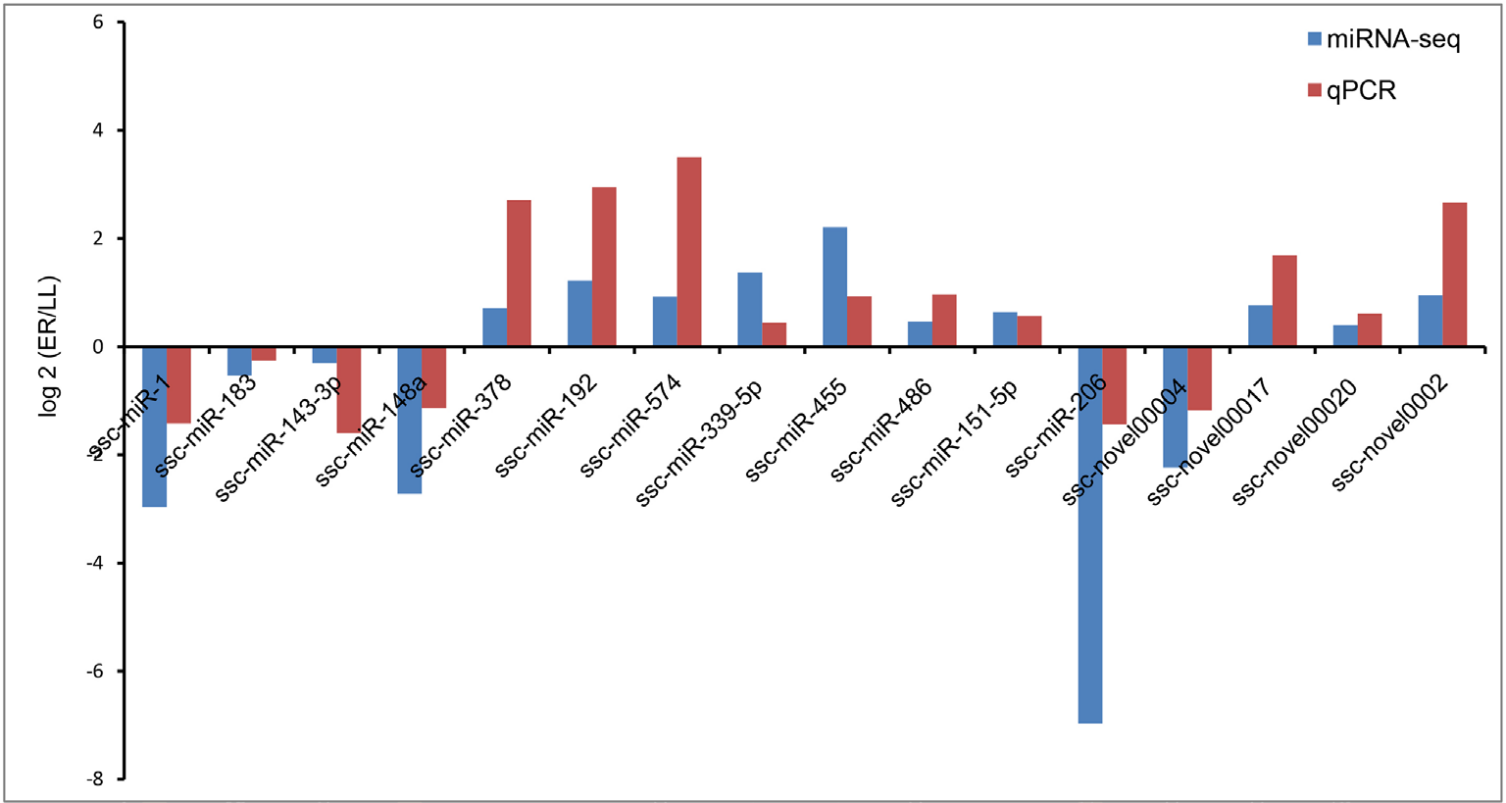
Validation of deep sequencing results *via* real-time qRT-PCR.

The stem-loop qRT-PCR assay was used to specifically detect mature miRNAs. U6 snRNA was selected as the reference gene. Sixteen miRNAs were chosen for validation by qRT-PCR and the primers used are listed in **Supplementary Table S1**. The expression patterns for the 16 miRNAs were consistent with those in sequencing data (**Figure 5**).

### Functional Annotation of DEMs in Endometrial Tissue Samples

**Figure 6:**
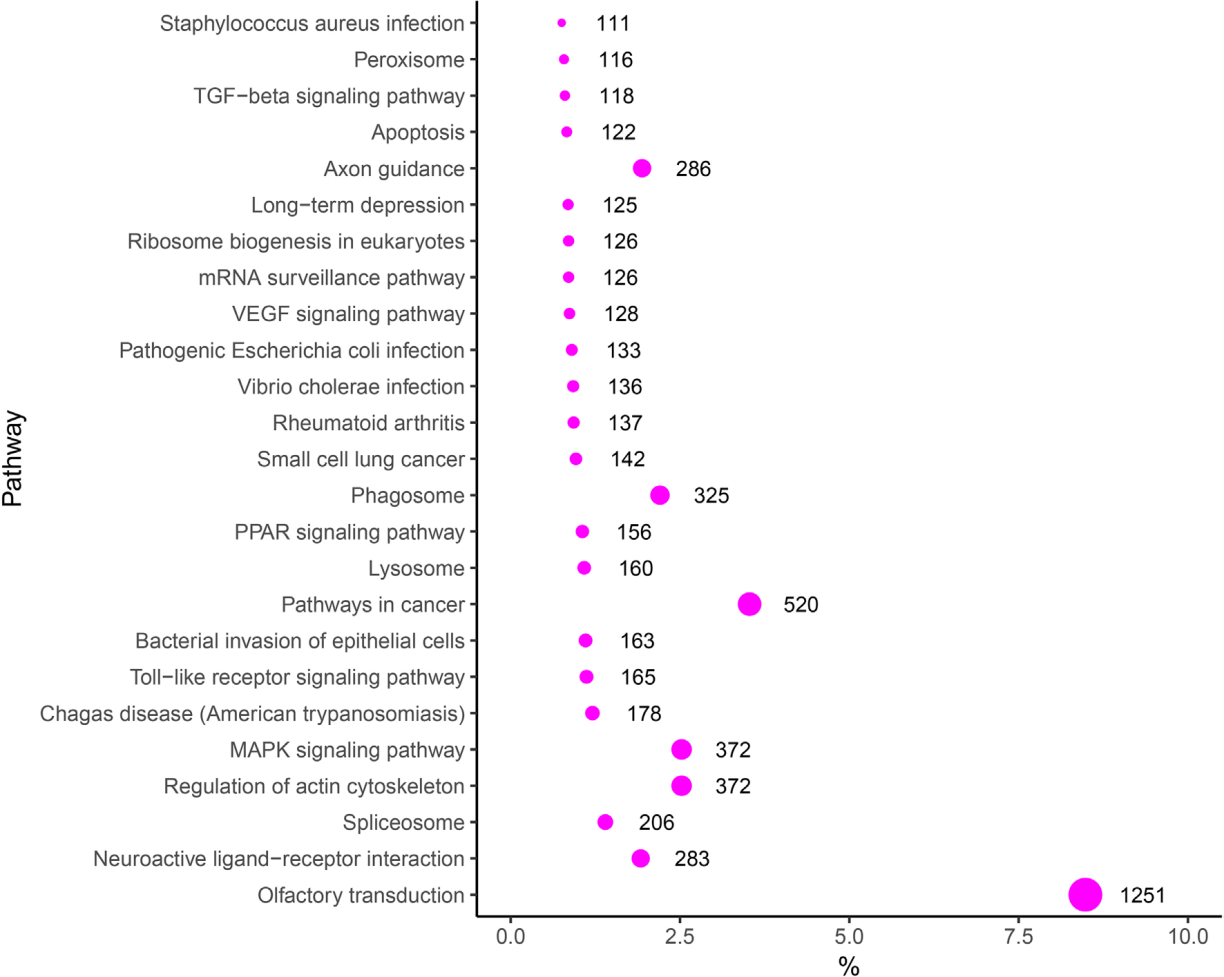
KEGG enrichment scatter plot of the targets of DEMs.

To evaluate the biological functions of these DEMs, target genes of DEMs were predicted by RNAhybrid and TargetScan, and the KEGG pathway analysis of these target genes was performed. Thus, 275 significantly enriched signaling pathways were obtained, such as with “VEGF signaling pathway” (Angiogenesis-related pathway), “Toll-like receptor signaling pathway” (Immune-related pathway), “Regulation of actin cytoskeleton” (Tissue remodeling-related pathway), and “MAPK signaling pathway, TGF-β signaling pathway and Apoptosis” (Proliferation and apoptosis) in the top 25 signaling pathways (**Figure 6**). In addition, some other target genes were annotated to reproduction-associated pathways, including “Steroid hormone biosynthesis,” “Progesterone-mediated oocyte maturation,” “Steroid biosynthesis,” “GnRH signaling pathway,” and “p53 signaling pathway.”

### miR-206 Directly Targeted 3′-UTR of IGF-1 and Inhibited its Protein Expression

**Figure 7:**
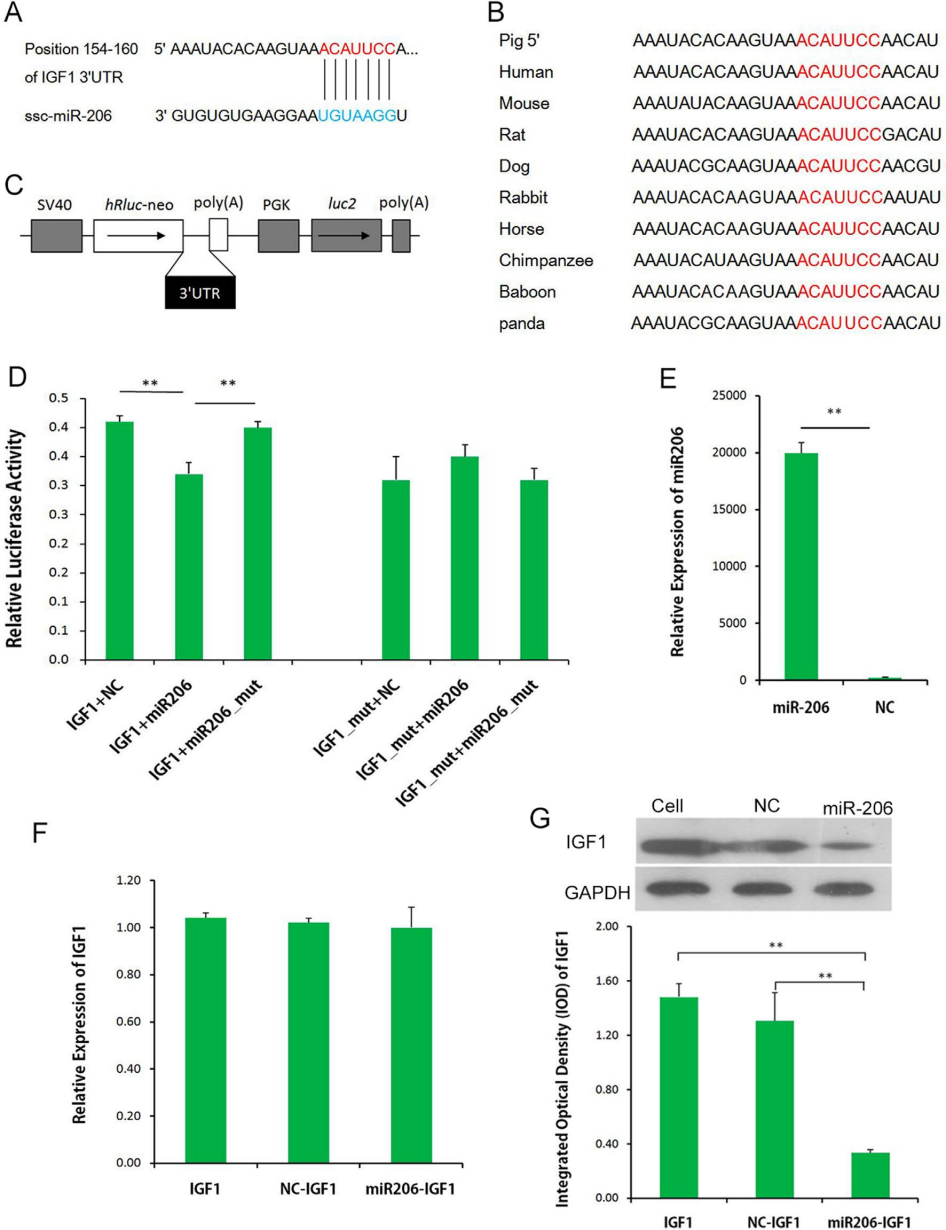
miR-206 targets the 3′-UTR of IGF-1. **(A)** Predicted binding site of miR-206 in the 3′-UTR of IGF-1. **(B)** Binding site of miR-206 is highly conserved among mammals. **(C)** IGF-1 3′-UTR was inserted into the pmirGLO dual-luciferase reporter vector at the 3′-end of the *Renilla* luciferase gene (hRluc). **(D)** IGF-1 3′-UTR or IGF-1 3′-UTR-Mutant construct was co-transfected with miR-206, miR-206_mut, or NC, as indicated, into PK cells, and normalized *Renilla* luciferase activity was determined. **(E)** Expression of miR-206 in the porcine skeletal muscle SCs infected with pri-miR-206 expression lentivirus or NC lentivirus. **(F)** Expression of IGF-1 mRNA in SCs and SCs infected with pri-miR-206 expression lentivirus or NC lentivirus. **(G)** Expression of IGF-1 protein in SCs and SCs infected with pri-miR-206 expression lentivirus or NC lentivirus. Results are mean ± SD (three independent replicates per group). *P < 0.05; **P < 0.01 (Student’s *t* test).

miR-206 was the top DEM, and it was expressed in both LL and ER pigs. To uncover the function of miR-206, the potential targets of miR-206 in the data bank (www.targetscan.org; **Figure 7A**) was examined, and IGF-1 was identified as a prime target, with a highly conserved complementary miR-206-binding site in its 3′-UTR across mammals from humans to pandas (**Figure 7B**). As shown in **Figure 7**, the dual-luciferase reporter assay system showed a significant reduction of the *Renilla*/firefly luciferase ratio in the wild-type miRNA mimic compared to the mutant-type group. However, miR-206 had no appreciable inhibitory effect on a mutated IGF-1 3′-UTR dual-luciferase construct (**Figures 7C, D**). These results demonstrate the specific inhibition of IGF-1 expression by miR-206. miRNA regulates gene expression at the transcriptional level or at translational levels. To determine the regulation mechanism of miR-206, qRT-PCR and Western blot analysis were performed. Although no significant inhibition was detected at the IGF-1 mRNA level in porcine skeletal muscle SCs that were infected with pri-miR-206 expression lentivirus (**Figures 7E, F**), the inhibitory effect of miR-206 on IGF-1 protein expression was determined by Western blot analysis (**Figure 7G**). Therefore, it was confirmed that miR-206 directly targeted 3′-UTR of IGF-1 and inhibited its protein expression but not its mRNA transcription.

## Discussion

The miRNA system is a huge regulatory network of cellular processes, with a single miRNA being able to post-transcriptionally silence multiple mRNAs, while each mRNA can be targeted by numerous miRNAs (Friedman et al., 2008; Lu and Clark, 2012). In humans, more than 30% of the mRNAs are predicted to be miRNA targets (Griffiths-Jones et al., 2007). Recently, some miRNAs were found to be associated with endometrial receptivity, embryo development, and implantation (Ariel et al., 2011; Liu et al., 2016). In the present study, the sRNA profiles of endometrial tissues from ER and LL swine endometrium on GD12 using sequencing technology were compared to understand the miRNA-mediated regulation of embryo implantation. Our studies revealed the differential expression of 96 known miRNAs and 68 novel miRNAs in ER and LL endometrium, and the identification of miRNAs and target genes may be useful to develop new techniques and strategies for improving embryonic survival during implantation.

### Highly Abundant miRNAs Might Affect Endometrial Remodeling

ssc-miR-143-3p, ssc-let-7a, and ssc-miR-21 were the top three miRNAs that were highly expressed in both LL and ER libraries. They were also found to be highly expressed in the endometrium of Meishan and Yorkshire pigs during early gestation in a recent published paper (Li et al., 2018). Mu et al. found that miR-143-3p inhibits proliferation and induced apoptosis in human hypertrophic scar fibroblast cells, and it also inhibited extracellular matrix production-associated protein expression (Mu et al., 2016). Several other studies also demonstrated that miR-143-3p suppressed proliferation and induced apoptosis in different carcinoma cells (He et al., 2016; Chen et al., 2017); thus, the highly expressed miR-143-3p in the porcine endometrium might also play a role in regulating the proliferation and apoptosis of endometrial cells. For let-7a, a functional investigation also revealed that it suppressed the proliferation of endometrial carcinoma (Liu et al., 2013b), and another study demonstrated that it markedly suppressed the proliferation, migration, and invasion of gastric cancer cells by down-regulating PKM2 (Tang et al., 2016). Furthermore, let-7a is involved in regulating the implantation process by the modulation of the expression of integrin-β3 and mucin 1 (Liu et al., 2012; Inyawilert et al., 2015).

In addition, miR-21 has been causally linked to cellular proliferation, apoptosis, and migration in a wide variety of cancers (Asangani et al., 2008; Frankel et al., 2008). Previous studies have suggested that miR-21 was involved in embryo implantation in mouse (Hu et al., 2008), and a recent study provided evidence that miR-21 expressed in extracellular vesicles is very important in preimplantation embryo development (Lv et al., 2018). The KEGG pathway analysis of common miRNAs indicated that the predicted target genes were enriched in 1) cell self-renewal and degradation, including “Apoptosis,” “Autophagy,” “Ubiquitin-mediated proteolysis,” “Longevity-regulating pathway,” and “AMPK signaling pathway”; 2) cell motility, including “Regulation of actin cytoskeleton,” “Focal adhesion,” “ECM-receptor interaction,” and “Rap1 signaling pathway”; and 3) cell proliferation and differentiation, including “FoxO signaling pathway,” “mTOR signaling pathway,” and “MAPK signaling pathway,” Based on these results, it can be inferred that most highly abundant miRNAs in porcine endometrium mainly played important roles in regulating endometrial remodeling at the time of implantation.

### Differentially Expressed Known miRNAs Related to Proliferation and Angiogenesis

Of the 96 differentially expressed known miRNAs, more than 80% were up-regulated in ER compared to LL sows; among them, ssc-miR-29c was the top miRNA that was expressed in both breeds (**Supplementary Table S7**). A recent study demonstrated that miR-29c affects human endometrial cells by suppressing cell proliferation and invasion as well as promotes cell apoptosis by inhibiting c-Jun expression (Long et al., 2015). It was also shown previously that miR-29c inhibited cell proliferation and induced apoptosis in many types of carcinoma cells (Wang et al., 2011; Liu et al., 2013a). Similarly, ssc-miR-214, which was among the top five miRNAs up-regulated in ER compared to LL sows (**Supplementary Table S7**), also played a role in promoting apoptosis and suppressing cell proliferation in several types of cells (Feng et al., 2011; Yang et al., 2013; Zhang et al., 2014). These results indicate that the markedly higher expression of miRNAs in ER than in LL (i.e., miR-29c and miR-214) could also play a key role in inhibiting endometrial cell proliferation and invasion, which could contribute to developing a more stable uterine environment. The results are also consistent with the authors’ previous findings, which also revealed that the endometrium of ER pigs had a lower growth-promoting ability (Zhang et al., 2013). Strong evidence has shown that increased prolificacy of Chinese Taihu pigs might be due to an increased embryonic survival resulting from the more stable uterine environment and increased uterine receptivity (Stroband et al., 1992; Youngs et al., 1993; Youngs et al., 1994).

Pathway analyses can provide a better understanding of the molecular functions and biological processes of target genes. Among the target genes of differentially expressed known miRNAs, some KEGG pathways that are important for reproduction were significantly enriched. Notably, the mitogen-activated protein kinase (MAPK) signaling pathway, the Toll-like receptor signaling pathway, the peroxisome proliferator-activated receptor (PPAR) signaling pathway, the vascular endothelial growth factor (VEGF) signaling pathway, and the transforming growth factor-β (TGF-β) signaling pathway were in the top 25 signaling pathways. The MAPK signaling pathway is involved in the regulation of human endometrial cell proliferation (Park et al., 2017; Zhang et al., 2018). In another study, research data indicated that the activation of the MAPK signaling pathway can increase the proliferation of porcine uterine LE cells and may affect implantation in early pregnancy in pigs (Lim et al., 2018). In addition, the PPAR and TGF-β signaling pathways were also related to cell proliferation and had influence on implantation (Lim and Dey, 2000; Li et al., 2004; Chang et al., 2008; Tsang et al., 2013; Cheng et al., 2017; Song et al., 2018). The Toll-like receptor signaling pathway is responsible for innate immune responses, and studies provide evidence that this pathway takes part in implantation by regulating trophoblast cells’ adhesion to endometrial cells (Montazeri et al., 2016). Studies provide evidence that the VEGF signaling pathway is known as the regulator of several endothelial cell functions, including mitogenesis, permeability, vascular tone, and the production of vasoactive molecules (Giles, 2001). Previous studies also indicated that it plays important roles in implantation and maintenance of pregnancy (Das et al., 1997; Halder et al., 2000; Möller et al., 2001; Hannan et al., 2011). Collectively, these pathway analyses illustrate some of the possible roles of highly expressed miRNAs in reproduction.

IGF-1 can regulate endothelial cell migration and promote angiogenesis (Shigematsu et al., 1999). In the human endometrium, it was found that IGF-1 participates in the maintenance of an angiogenic phenotype by inducing VEGF expression (Bermont et al., 2000). Furthermore, a recent study reported that IGF-1 is a critical determinant of neonatal porcine uterine development (George et al., 2018). Our results demonstrated that IGF-1 protein expression was directly inhibited by miR-206, which were highly expressed in LL and lowly expressed in ER. This suggests that the low expression of miR-206 in ER might facilitate the angiogenesis of endometrium during peri-implantation, but further studies are required to verify this hypothesis.

## Conclusions

In summary, Illumina sequencing was used to identify 288 distinct miRNAs, consisting of 202 previously reported and 86 novel miRNAs, from porcine endometrium in two different reproduction capacity breeds. In a comparison of ER to LL sows, 96 significantly differentially expressed known miRNAs (78 up-regulated and 18 down-regulated) were identified. The target gene expression and pathway enrichment analyses indicated that these DEMs may influence embryonic implantation by regulating pathways related to proliferation, immunization, and angiogenesis. Our findings help gain a better understanding of the role of miRNAs in the regulation of embryonic implantation and embryonic survival in pigs. Future studies to identify target mRNAs regulated by abundant miRNAs in the endometrium using a single type of endometrial cell (i.e., luminal or glandular epitheliums) will be critical to uncover their exact biological functions.

## Data Availability

The datasets used and analysed during the current study are available from the corresponding author on reasonable request. The raw reads produced in this study were deposited in the NCBI Sequence Read Archive (SRA), available using accession number PRJNA50573.

## Ethics Statement

All researches involving animals were conducted according to animal ethics guidelines and approved by the Animal Care and Use Committee of South China Agricultural University (Guangzhou, China).

## Author Contributions

JL, ZW, and HZ designed the study. LH, RL, XQ, and SW performed the experiments, analyzed the data, and drafted the manuscript. XW performed the sequencing analysis. All authors read and approved the final manuscript.

## Funding

This work was supported by the National Natural Science Foundation of China (31802033), the Guangdong Provincial Promotion Project on Preservation and UtiIization of Local Breed of Livestock and Poultry (4300-F18260), and the Science & Technology Planning Project of Guangzhou in China (201904010434). The funders had no role in study design, data collection and analysis, decision to publish or preparation of the manuscript.

## Conflict of Interest Statement

The authors declare that the research was conducted in the absence of any commercial or financial relationships that could be construed as a potential conflict of interest.

## Supplementary Material

Supplementary Figure 1 ClueGo network of pathways. Each node represents a pathway. The enrichment significance of pathway is reflected by the size of the nodes. Node color, represents the class that they belong. Mixed coloring means that the specific node belongs to multiple classes.

Supplementary Table 1 Primer sequences for qPCR.

Supplementary Table 2 The detailed sequence information in ER and LL small RNA libraries.

Supplementary Table 3 Match_hairpin in ER library.

Supplementary Table 4 Match_hairpin in LL library.

Supplementary Table 5 Sequence editing of bases in the seed region of the miRNAsin ER and LL.

Supplementary Table 6 Global expression profile of endometrial miRNAs on day 12 of gestation in LL and ER pigs.

Supplementary Table 7 The differentially expressed miRNAs in ER and LL small RNA libraries.
