# Supplementary figures and images for: Differential microRNA Expression in Porcine Endometrium Involved in Remodeling and Angiogenesis That Contributes to Embryonic Implantation
Source: Front Genet. 2019 Jul 26;10:661. doi: 10.3389/fgene.2019.00661 (PMC6677090; doi:10.3389/fgene.2019.00661)

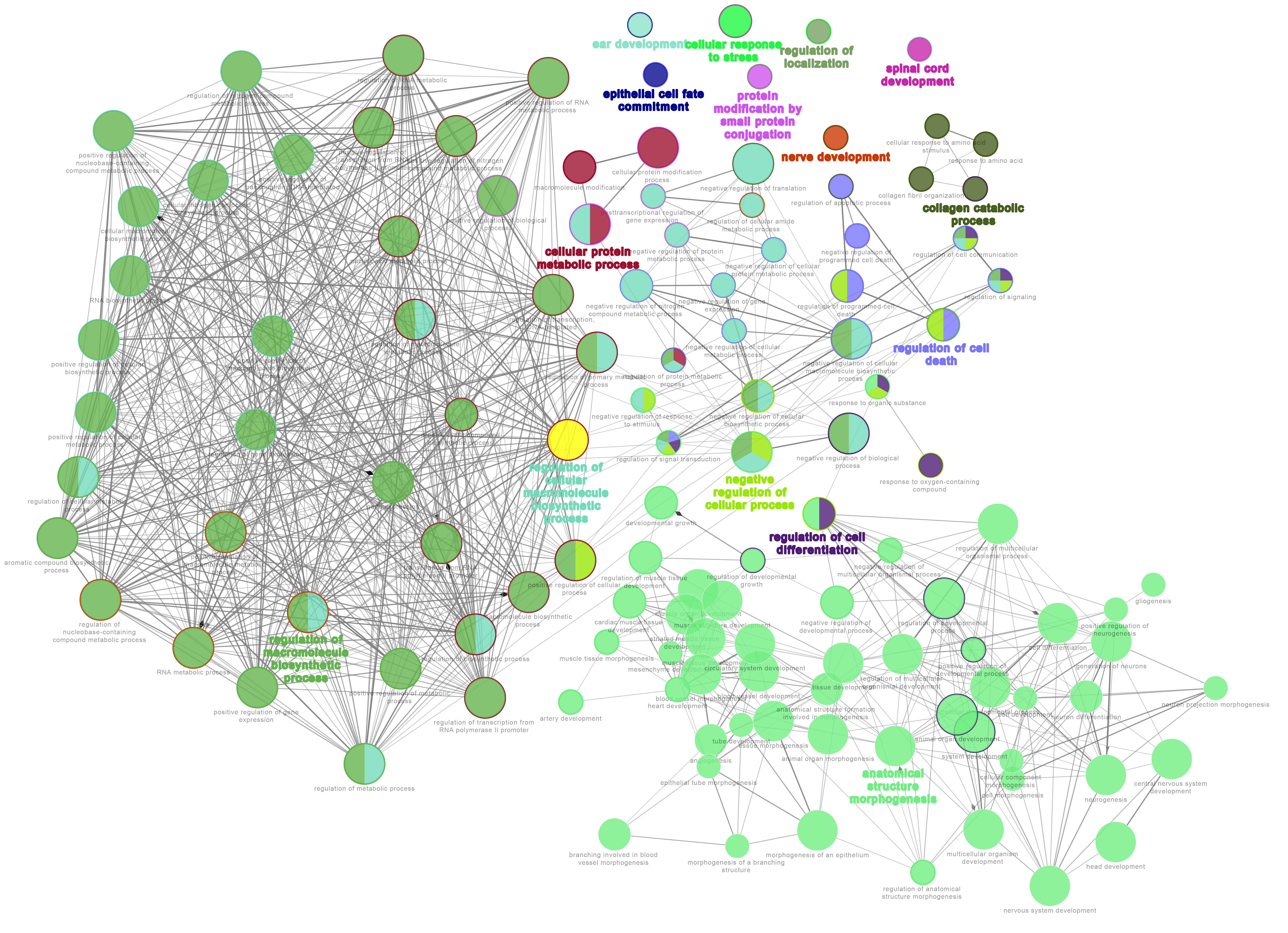

Supplement: Supplementary Figure 1 — ClueGo network of pathways. Each node represents a pathway. The enrichment significance of pathway is reflected by the size of the nodes. Node color, represents the class that they belong. Mixed coloring means that the specific node belongs to multiple classes. [file Image_1.jpg]
